# Stem albatrosses wandered far: a new species of *Plotornis* (Aves, Pan-Diomedeidae) from the earliest Miocene of New Zealand

**DOI:** 10.1080/03036758.2023.2266390

**Published:** 2023-11-13

**Authors:** Daniel T. Ksepka, Alan J. D. Tennyson, Marcus D. Richards, R. Ewan Fordyce

**Affiliations:** a Bruce Museum, Greenwich, CT, USA; b Museum of New Zealand Te Papa Tongarewa, Wellington, New Zealand; c Department of Geology, University of Otago, Dunedin, New Zealand

**Keywords:** Procellariiformes, Aves, Cenozoic, fossil, phylogeny

## Abstract

Albatrosses are among the most intensely studied groups of living birds, yet their fossil record remains sparse. Despite modern albatrosses being more abundant and widespread in the Southern Hemisphere, the vast majority of fossil albatrosses identified to date come from Northern Hemisphere localities. Here, we describe *Plotornis archaeonautes* sp. nov., a new albatross species from the earliest Miocene that represents the earliest record of Procellariiformes in New Zealand and the earliest uncontroversial record of the clade Pan-Diomedeidae from the Southern Hemisphere. Phylogenetic analyses support the placement of *Plotornis* outside of the clade uniting all extant albatrosses. The new fossil reveals that stem lineage albatrosses were widespread by the onset of the Neogene. Although the humerus of *Plotornis archaeonautes* exhibits a short processus supracondylaris dorsalis, this early species may have possessed at least one of the unique ossifications associated with the patagial bracing system present in modern albatrosses.

## Introduction

Albatrosses are perhaps the most iconic seabirds, and have long featured prominently in both cultural works and scientific research on avian ecology. Despite their predominantly southern present-day distribution, albatrosses are represented by only a handful of Southern Hemisphere fossils. The oldest putative southern records of albatrosses are fragmentary specimens from the Eocene La Meseta Formation of Seymour Island, Antarctica. Acosta Hospitaleche and Gelfo (2017) named *Notoleptos giglii* based on a nearly complete but damaged tarsometatarsus. Mayr and Tennyson (2020), however, considered the affinities of this fossil to be uncertain. We agree with this assessment, especially given the small size and the fact that the hypotarsal crests are not preserved. Two other putative records from the La Meseta Formation were reported in earlier work but not illustrated at the time: an incomplete tarsometatarsus (Tambussi and Tonni 1988) and the rostral tip of a beak (Noriega and Tambussi 1996; see also Tambussi and Acosta Hospitaleche 2007). Both of these fossils were considered ?Diomedeidae indet. by Acosta Hospitaleche and Gelfo (2017). As with *Notoleptos giglii*, support for assignment of these fossils to Diomedeidae should be considered tentative pending discovery of more complete material. The oldest putative record of Diomedeidae from New Zealand is a furcula from the late Oligocene Kokoamu Greensand described as *Manu antiquus* by Marples (1946). However, Olson (1985) noted the furcula was dissimilar to that of extant albatrosses and Mayr (2009) subsequently suggested this fossil may represent Pelagornithidae.

Setting aside these uncertain records, all Southern Hemisphere albatross fossils come from Neogene deposits, and most are highly incomplete (Wilkinson 1969; Olson 1983, 1984, 1985; Walsh and Hume 2001). One exception is the small stem albatross *Aldiomedes angustirostris*, which is known from a complete skull from the late Pliocene Tangahoe Formation of New Zealand. This species differs from extant albatrosses in the narrower beak, larger nares and more well-developed temporal fossae (Mayr and Tennyson 2020).

In contrast to their scarcity in the Southern Hemisphere, fossil albatrosses are well-represented in Oligocene-Pleistocene Northern Hemisphere localities (Milne-Edwards 1874; Lydekker 1886; Brodkorb 1963; Howard 1966, 1968; Olson and Rasmussen 2001; Davis 2003; Dyke et al. 2007; Mayr and Goedert 2017). This fossil distribution reflects a wider past geographical range for albatrosses, but almost certainly also reflects sampling biases favouring European and North American shallow marine deposits. The oldest uncontroversial Northern Hemisphere albatross fossil is the holotype of *Tydea septentrionalis* from the early Oligocene of Belgium (Mayr and Smith 2012). Although *Tydea septentrionalis* fell within the range of sizes spanned by modern albatrosses, many Oligocene-Miocene fossil albatrosses were smaller birds. *Diomedavus knapptonensis* from the late Oligocene of Washington state (USA) falls below the size of the smallest extant albatrosses, and was identified as a stem albatross by Mayr and Goedert (2017). A slightly larger albatross was also identified from the early/middle Miocene Astoria Formation of Washington state (USA), but due to non-overlapping elements was not assigned to any existing genus (Mayr and Goedert 2017).

*Plotornis* was the first fossil albatross taxon to be formally named and has a complicated taxonomic history. The type species *Plotornis delfortrii* was based on a partial humerus and tarsometatarsus from the Early Miocene (MN 2-3; Mlíkovsky 2002) of Léognan, France. Milne-Edwards (1874) identified this species as a relative of albatrosses. Lambrecht (1933) referred *Plotornis delfortrii* to Procellariidae, a perplexing decision given that his brief summary noted the fossils resembled modern *Diomedea*. Olson (1985) re-assigned *Plotornis* to Diomedeidae, and questioned whether *Plotornis* might be better merged with *Diomedea*, the single genus into which all extant albatrosses were generally lumped at the time.

The second named species of *Plotornis* is no longer considered to represent an albatross. Milne-Edwards (1869–1874, p. 572) coined the name *Puffinus arvernensis* without a description, creating a *nomen nudum*. Shufeldt (1896) rendered the name *Puffinus arvernensis* available by publishing illustrations of a fossil tarsometatarsus provided to him by Milne-Edwards. Nearly a century later, Cheneval (1984) referred a pathological coracoid to *Puffinus arvernensis,* and transferred the species to the genus *Plotornis*. However, Mayr and Smith (2012) argued that neither the tarsometatarsus nor the coracoid belong to an albatross. Shortly thereafter, Mayr and Pavia (2014) re-studied the putative anseriform ‘*Chenornis*’ *graculoides*, which was named by Portis (1884) based on a partial skeleton from the early Miocene of Italy, and convincingly reclassified it as *Plotornis graculoides*. Most recently, Ksepka (2014) referred a few bones from the Oligocene Chandler Bridge Formation of South Carolina (USA) to *Plotornis*.

All potential crown albatrosses come from Neogene deposits. Several Miocene albatross fossils have been assigned to the extant genus *Diomedea* (today reserved for the great albatrosses), including *Diomedea milleri* and *Diomedea californica* from California, *Diomedea tanakai* from Japan and *Diomedea thyridata* from Australia. However, all of these species are known from scant material and it remains uncertain which if any are in fact close relatives of the great albatrosses (Mayr and Smith 2012). Although albatrosses occur only as vagrants in the North Atlantic today, fossil albatrosses are particularly diverse and abundant in the Pliocene Yorktown Formation of North Carolina (USA), where Olson and Rasmussen (2001) recognised five species, all of which they assigned to the extant genus *Phoebastria*. Three of these species were considered to potentially represent ancestors of the extant *Phoebastria albatrus, Phoebastria immutabilis* and *Phoebastria nigripes*, whereas the other two were assigned to the extinct species *Phoebastria anglica* (also known from the Pleistocene of England and Miocene/Pliocene of Florida; Wetmore 1943; Dyke et al. 2007) and *Phoebastria rexsularum. Phoebastria albatrus* persisted into the Pliocene-Pleistocene in Morocco (Mourer-Chauviré and Geraads 2010; Mourer-Chauviré 2016) and the Pleistocene (∼400 ka) of Bermuda (Olson and Hearty 2003). This remarkable diversity in an area now devoid of albatrosses documents that the clade vanished from the North Atlantic only recently (Olson and Rasmussen 2001; Olson and Hearty 2003).

## Geological setting

**Figure 1.**
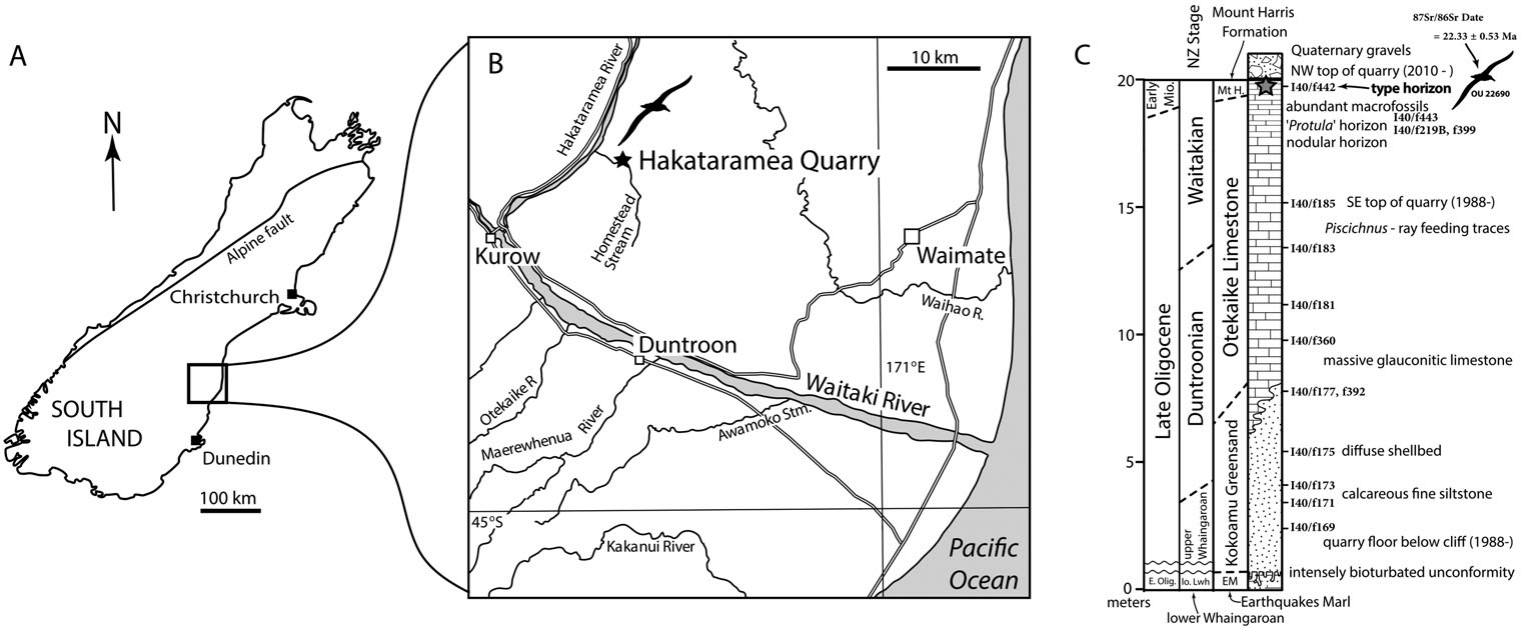
**A**, Map of the South Island of New Zealand with **B**, location and **C**, stratigraphy of Hakataramea Quarry with holotype horizon indicated. Images and data modified from Boessenecker and Fordyce (2017), Fordyce (2009), Gottfried et al. (2012) and Hornibrook (1996), and updated using authors’ REF and MDR field data (pers. obs. 1987–present). New Zealand Fossil Record File Database numbers (www.fred.org.nz) are included to tie into previous fossils described from site. Note that the fossil positions are approximate as stratigraphic position is complicated by the actively changing quarried face, the strata is massive with rare marker beds, and there is a stratigraphic dip ∼7° NW.

An associated partial *Plotornis* skeleton was discovered in 2010 during a field trip led by R. Ewan Fordyce. The fossil was collected from the upper back bench on the northwest wall of Hakataramea Quarry (referred to as Haughs’ Quarry, Hurstlea Quarry or Hakataramea Lime Quarry in some references), an active quarry in the Hakataramea River valley of South Canterbury, South Island, New Zealand (Fordyce and Maxwell 2003; Gottfried et al. 2012; Meadows et al. 2015; Boessenecker and Fordyce 2017) (Figure 1). The albatross specimen (OU 22690; field number REF 13.7.10.2) was discovered incidentally during the excavation of a mysticete whale skeleton (OU 22918, cf. *Mauicetus*; field number REF 6.6.10.3). All of the holotype elements were collected within a single block of glauconitic muddy limestone except for a fragment of the left ulna, which was found as float nearby and is presumed to have eroded free prior to discovery.

The horizon in question represents the uppermost marine beds exposed at Hakataramea Quarry, which are referred to the Mount Harris Formation. Both the Otekaike Limestone and Mount Harris Formations are exposed at the quarry. The exposure of the Otekaike Limestone at the quarry is limited to the massive Maerewhenua member of Gage (1957) and forms a condensed late Oligocene sequence compared to localities in the Waitaki Valley, being only ∼12 m thick and reflecting a mid-continental shelf, neretic setting distant from open ocean (as shown by a distinct lack of adult planktic foraminiferans) but also very little terrigenous input from a presumed low lying landmass to the west. The top of the formation grades into muddy glauconitic limestone of the Mount Harris Formation (see Fordyce 2009; Tanaka and Fordyce 2015, 2016), and is in turn overlain uncomformably by Pleistocene gravels. *Lentipecten* scallop shells associated with OU 22918 and OU 22690 produced an error weighted mean ^87^Sr/^86^Sr ratio of 0.708294 ± 0.000028 (2 standard error) which equates to an age of 22.33 ± 0.53 Ma (Marx et al. in press). Matrix sampled directly from the holotype block yielded specimens of the planktic foraminifera *Globoturborotalita connecta* ( =  ‘*Globigerina*’ *connecta*). The first appearance of *G. connecta* indicates the base of the upper Waitakian zone of Jenkins (1965; see Hornibrook et al. 1989). The timing of this bioevent can be constrained by revised dates from previous Sr isotpe data of Morgans et al. (1999) and Graham et al. (2000) to a possible range of ∼22.0–22.7 Ma (McArthur et al. 2020: data LOWESS 6C 16_03_2020; Marx et al. in press). The biostratigraphic and isotope data therefore support assignment of the type horizon to the upper Waitakian local stage and thus an earliest Miocene (Aquitanian) age for the fossil. A partial radius and tarsometatarsus were collected as float by Jana Wold and Alan Tennyson. These bones cannot be placed with as high degree of stratigraphic precision as the associated skeleton, and could potentially be from either the same fossiliferous horizon or from older late Oligocene deposits of the Otekaike Limestone.

## Institutional abbreviations

AMNH – American Museum of Natural History (Ornithology Collections), New York NY, USA; NMNZ – Museum of New Zealand Te Papa Tongarewa, Wellington, New Zealand OM – Otago Museum, Dunedin, New Zealand; OU – Otago University Geology Museum, Dunedin, New Zealand; OUVC – Ohio University Vertebrate Collections; USNM – National Museum of Natural History, Smithsonian Institution, Washington DC, USA; YPM – Yale Peabody Museum of Natural History, New Haven CT, USA.

## Systematic palaeontology

Aves Linnaeus, 1758

Procellariiformes Fürbringer, 1888

Pan-Diomedeidae G.R. Gray, 1840

*Plotornis* Milne-Edwards 1874

**Type Species.** *Plotornis delfortrii* Milne-Edwards 1874

**Included Species.** *Plotornis delfortrii*, *Plotornis graculoides* and *Plotornis archaeonautes* (sp. nov.)

**Revised Diagnosis**. *Plotornis* is differentiated from all crown albatrosses by a more slender beak and conspicuously shorter processus supracondylaris dorsalis which tapers distally (distal margin squared in crown albatrosses). Differs from *Tydea* in having a longer processus supracondylaris dorsalis which is cranial-laterally directed (proximally deflected in *Tydea*). Differs from *Diomedavus* in more proximally located apex of crista deltopectoralis of the humerus, and in the absence of an ossified retinaculum extensorium of the tarsometatarsus. Differs from *Aldiomedes* in wider beak. *Plotornis* differs from poorly known taxa historically lumped in the genus *Diomedea* as follows: from ‘*Diomedea’ thyridata* and potentially also ‘*Diomedea’ tanakai* in having a more slender beak tip (the wide beak tip in the latter is based on a tentatively referred specimen); from ‘*Diomedea’ tanakai* in having a less medially flared rim of the cotyla medialis; from ‘*Diomedea’ californica* based on much smaller size (overall size approximately two-thirds that of *D. californica* based on proximal tarsometatarsus width); and from ‘*Diomedea’ milleri* in having a larger plantar opening of the lateral foramen vasculare proximale.

**Comments**. Pan-Diomedeidae is used here as a name for the albatross total group, i.e. all taxa more closely related to Diomedeidae than to any of the other extant clades of Procellariiformes.

*Plotornis archaeonautes* sp. nov.

urn:lsid:zoobank.org:act:44CDC41D-5249-4D12-9FBA-3783C10F3C32

**Figure 2.**
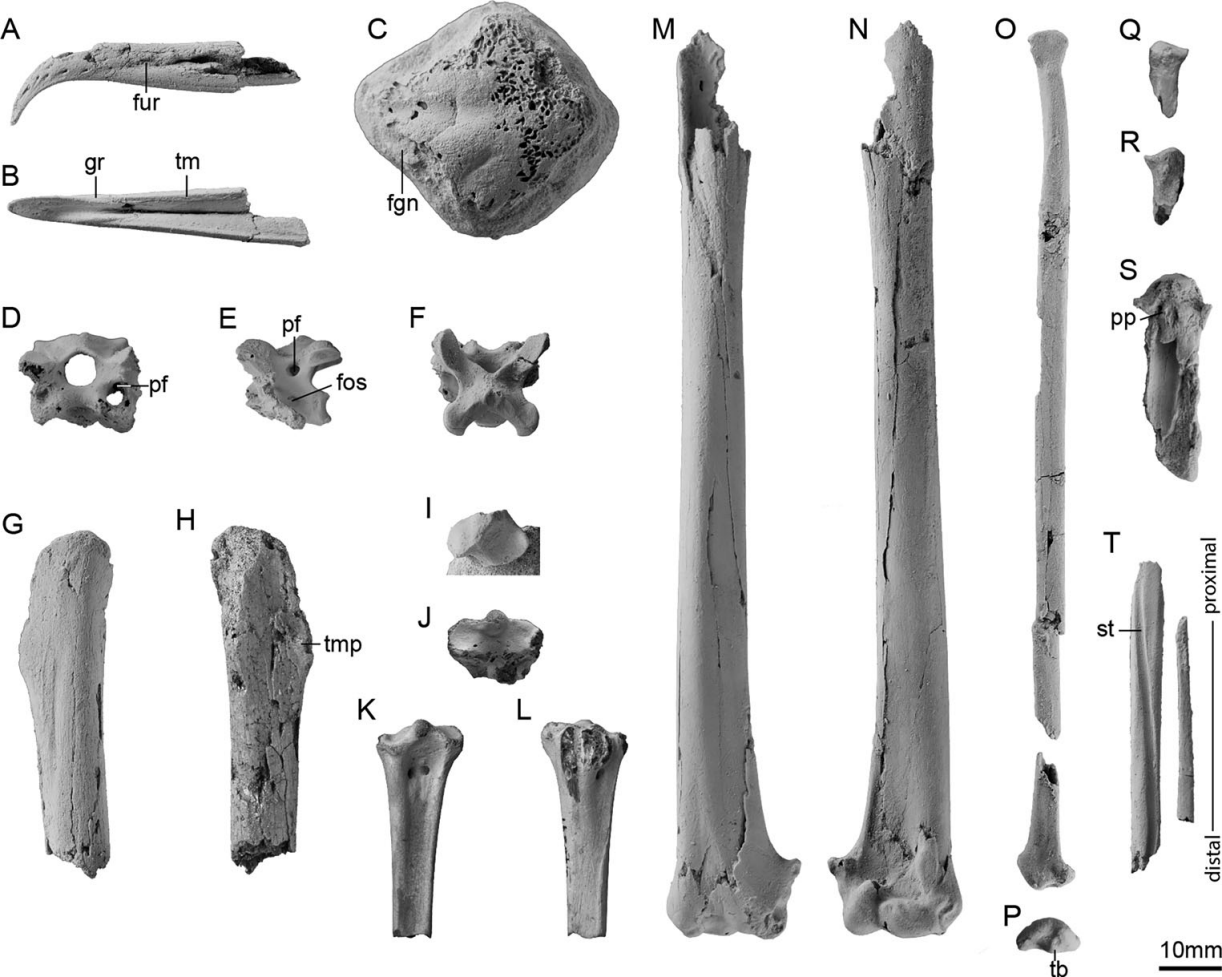
Holotype (OU 22690: A-I, M-P, S-T) and referred specimens (NMNZ S48291: J-L, NMNZ S47315: Q-R) of *Plotornis archaeonautes*. Beak tip in **A**, lateral and **B**, ventral views, **C**, skull roof in dorsal view, cervical vertebra 12 in **D**, cranial, **E**, lateral and **F**, dorsal views, fragment of left humerus in **G**, caudal and **H**, cranial views, **I**, proximal end of right ulna in proximal view, referred right tarsometatarsus in **J**, proximal, **K**, dorsal and **L**, plantar views, right humerus in **M**, caudal and **N**, cranial views, left radius in **O**, ventral (rotated slightly) and **P**, distal views, referred left radius in **Q**, ventral and **R**, dorsal views, **S**, proximal portion of right carpometacarpus in ventral view, **T**, shaft of left carpometacarpus in dorsal view. Abbreviations: **fgn**: fossa glandulae nasalis; **fos**: fossa housing subdivided pneumatic foramen; **fur**: furrow on lateral face of beak, **gr**: groove formed where furrow meets tomial margin; **pf**: pneumatic foramen; **pp**: processus pisiformis; **st**: sulcus tendineus; **tb**: tubercle at distal end of radius; **tm**: tomial margin; **tmp:** tubercle for the insertion of m. pectoralis.

**Holotype**. OU 22690: partial skull, cervical vertebra, right humerus lacking proximal end, shaft of left humerus, nearly complete left radius, proximal end of right and left ulna, proximal end of right carpometacarpus, shaft of left carpometacarpus (Figure 2).

**Referred material**. NMNZ S48291: proximal portion of right tarsometatarsus; NMNZ S47315 proximal portion of left radius (Figure 2). The tarsometatarsus is in the range expected for the species based on the proportions of *Plotornis delfortrii*. The referred radius does not differ meaningfully from the holotype but is ∼20% larger. This is within the range of size variation observed in extant albatross species, though more complete material may eventually reveal the presence of multiple albatross species in the fauna.

**Etymology**. From the Greek ἀρχαῖος (ancient) + ναύτης (mariner). The name reflects the ancient age and the wide oceanic range of *Plotornis* albatrosses, and makes allusion to the fabled albatross of Samuel Taylor Coleridge's The Rime of the Ancient Mariner.

**Type Locality and Horizon**. Earliest Miocene (Aquitanian) Mount Harris Formation, Hakataramea Quarry, South Canterbury, New Zealand (New Zealand Fossil Record Number I40/f442).

**Measurements**. Humerus distal width 19.5 mm (*Plotornis delfortrii:* ∼18.0 mm; *Plotornis graculoides*: 19.0 mm), tarsometatarsus proximal width 14.0 mm as preserved, estimated 15.0 mm when complete (*Plotornis delfortrii:* 12.6 mm; *Plotornis graculoides*: ∼14.5 mm).

**Diagnosis**. *Plotornis archaeonautes* differs from *Plotornis delfortrii* in the more proximodistally elongated tuberculum supracondylare ventrale, shallower fossa m. brachialis, and less ventrally deflected proximal tip of the condylus dorsalis, and differs from *Plotornis graculoides* in having a more weakly developed tubercle at the distal end of the crista deltopectoralis, a less widened proximal end of the tarsometatarsus (relative to shaft width), and a more distal origin of the crista plantaris lateralis of the tarsometatarsus.

**Comments**. Due to the incomplete nature of previously described material, our referral of the new fossil to *Plotornis* relies primarily on similarities in the distal end of the humerus. While it is possible that future discoveries will reveal differences requiring the erection of a new genus, we opt for a taxonomically conservative approach.

**Figure 3.**
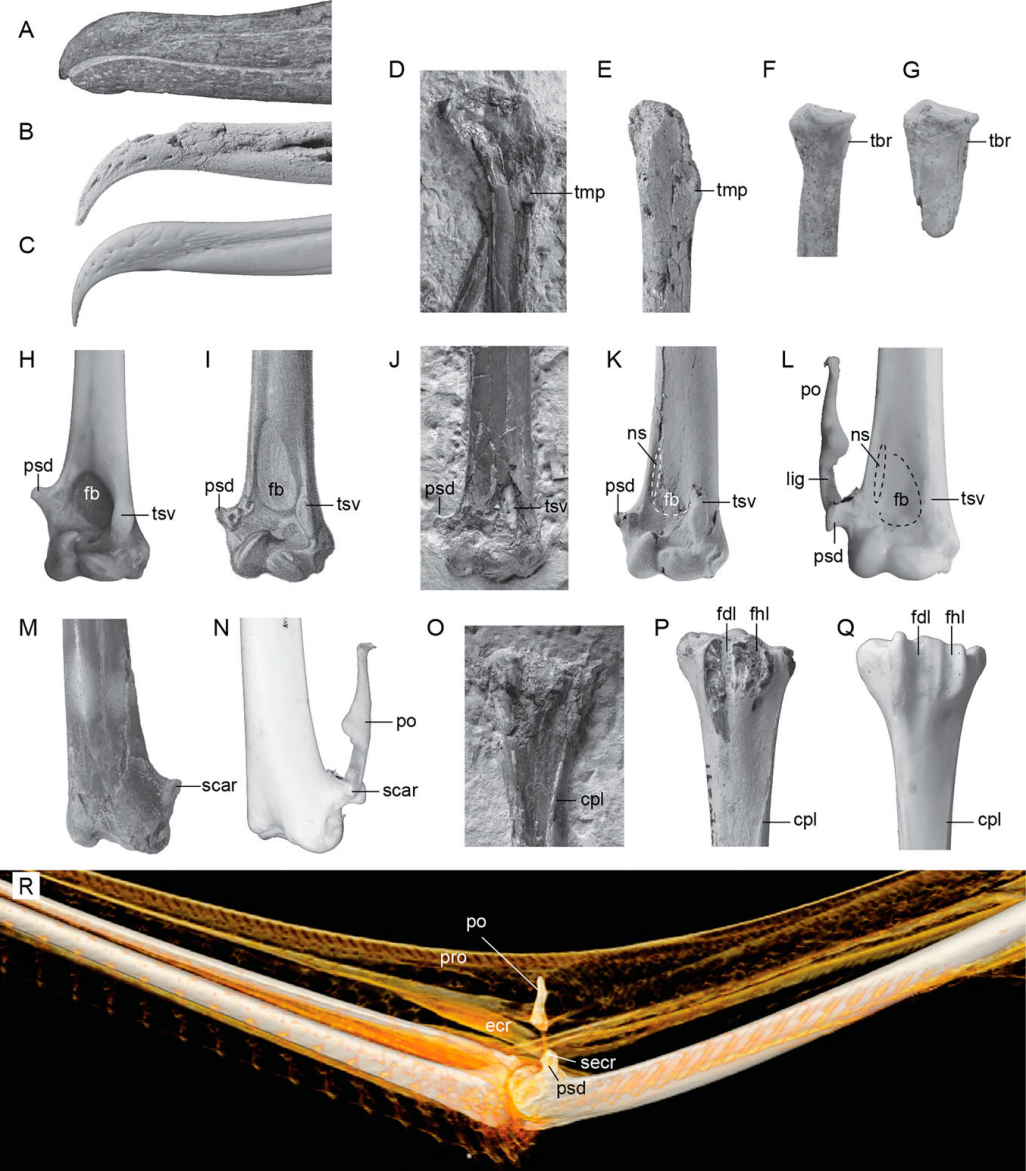
Comparison of the key features in fossil and extant albatrosses. Beak tip **A**, *Aldiomedes angustirostris* (holotype), **B**, *Plotornis archaeonautes* (holotype) and **C**, *Thalassarche carteri* (NMNZ OR22417). Proximal end of the humerus (cranial view) in **D**, *Plotornis graculoides* (holotype, mirrored for comparison) and **E**, *Plotornis archaeonautes*. Proximal end of the radius in **F**, *Plotornis archaeonautes* holotype and **G**, tentatively referred specimen (NMNZ S47315). Distal end of the humerus (cranial view) in **H**, *Macronectes giganteus* (OM AV10446), **I**, *Plotornis delfortrii* (holotype, image from Milne-Edwards 1874), **J**, *Plotornis graculoides*, **K**, *Plotornis archaeonautes* and **L**, *Thalassarche cauta steadi* (OM AV10444). Distal end of the humerus (dorsal view) showing attachment of the patagial ossicle in **M**, *Plotornis archaeonautes* and **N**, *Thalassarche cauta steadi*. Hypotarsal region in **O**, *Plotornis graculoides*, **P**, *Plotornis archaeonautes* and **Q**, *Phoebetria palpebrata* (NMNZ OR13310). **R**, CT scan of the wing of *Phoebetria immutabilis* (OUVC 10857) showing the relationships of the patagial ossicle and sesamoideum m. extensoris carpi radialis to the muscles and ligaments of the wing. Abbreviations: **cpl**: crista plantaris lateralis; **ecr**; m. extensor carpi radialis; **fb**: fossa m. brachialis; **fdl**: groove for tendon of m. flexor digitalis longus; **fhl**: groove for tendon of m. flexor hallucis longus; **lig**: ligament; **ns**: narrow scar bordering fossa m. brachialis; **po**: patagial ossicle; **pro**: ligamentum propatagiale; **psd**: procesus supracondylaris dorsalis; **scar**: scar for attachment of ligament connecting processus supracondylaris dorsalis and patagial sesamoid; **secr**; os sesamoideum m. extensoris carpi radialis; **tbr**: tuberculum bicipitalis radialis; **tmp:** tubercle for the insertion of m. pectoralis; **tsv**: tuberculum supracondylare ventrale. Photo L and N courtesy of On Lee Lau, © Tūhura Otago Museum Collection. CT rendering courtesy of Larry Witmer and Emily Caggiano (Ohio University). Images not to scale.

**Description**. As in extant albatrosses, the beak is strongly hooked and ends in a sharp point. It is however more slender and less dorsoventrally tall, and also less steeply downturned at the tip (Figure 3A–C). A narrow furrow extends along the lateral surface of the beak. Near the rostral tip, this furrow widens and becomes shallower as it deflects to end at the ventral margin of the premaxilla, creating a groove that interrupts the sharp ridge that forms the tomial margin (Figure 2B). In cross-section, the tomial margin of the premaxilla has a sharp lateral edge and slants smoothly towards the midline. This conforms to the shape of the tomial margin in extant albatrosses but contrasts with the condition in petrels, in which the cross-section of the tomial margin is concave, with a sharp lateral edge and a second sharp but less projected medial edge.

A portion of the calvarium is preserved separate from the beak. The bone surface of the skull roof is abraded, which makes it difficult to determine the depth of the fossae temporales. However, the intact portion indicates they were widely separated at midline as in extant albatrosses. Two sagittally oriented, ovoid convexities indicate the position of the eminentia sagittalis of the cerebrum, which are well-developed as in most Procellariiformes. A small intact shelf of bone along the left rim of the orbit indicates that the fossa glandulae nasalis was bounded laterally by a shelf of bone.

A single vertebra is identified as cervical vertebra 12 based on comparisons to extant albatrosses. Three pneumatic foramina pierce the vertebra on each side. One opens within a fossa on the lateral surface of the corpus, and is subdivided by a strut of bone. A second larger foramen pierces the lateral face of the neural arch, spanning the space between the zygapophysis cranialis and zygapophysis caudalis and reducing the arch to a thin lamina along its dorsal rim. The third foramen opens along the cranio-ventral face of the base of the zygapophysis cranialis. Pneumatic foramina are present in these positions in extant albatrosses, but show significant individual variation in development, occasionally even being present on one side and absent on the other side of a single vertebra. The neural spine is reduced to a low, subdivided tubercle that is continuous with two weak, posterolaterally directed ridges.

Only a small portion of the left humerus is preserved, which includes the base of the crista deltopectoralis. A tubercle for the insertion of m. pectoralis is present at the distal margin of the crista deltopectoralis, but is less strongly developed than in *Plotornis graculoides* (Figure 3D–E). The right humerus is well-preserved, but lacks the proximal end. The shaft is moderately cranio-caudally compressed as in most Procellariiformes (more strongly compressed in diving taxa such as *Puffinus*, some *Ardenna*, and *Pelecanoides*). At midshaft, the humerus has an ovoid cross section, but near the distal end the dorsal margin narrows to a sharp edge, which is a derived feature of albatrosses. As in extant Diomedeidae the fossa m. brachialis is shallow and subtriangular with weakly defined margins, contrasting with the much deeper circular pit present in Hydrobatidae, most Procellariidae, and the extinct Diomedeoididae. This fossa appears similarly shallow in *Plotornis graculoides* (Mayr and Pavia 2014: Figure 2D), but is depicted as more well-defined in *Plotornis delfortrii* in the illustration provided by Milne-Edwards (1874) (see Figure 3I–K). A moderately projected processus supracondylaris dorsalis is present, differing from that of crown albatrosses in its smaller size and tapering, rather than squared, distal end. On the dorsal face of the process, a slight depression is developed. Based on comparisons to extant albatross specimens, this depression represents the attachment surface for a ligament that tethers a sesamoid ossification. The elongate tuberculum supracondylare ventrale extends proximally beyond the level of the processus supracondylaris dorsalis, as in extant albatrosses but in contrast to other Procellariiformes. The dorsal face of the epicondylus dorsalis lacks part of its caudal border, but a deep depression marking the origin of m. entepicondylus ulnaris is partially preserved. The ventral face of the epicondylus ventralis bears a deep, round, subdivided depression corresponding to the origin of m. entepicondylus ulnaris. The sulcus scapulotricipitalis is shallow and the fossa olecrani is weakly developed.

The holotype radius is nearly complete. The tuberculum bicipitale radii is large and rounded. At the distal end a small, distally directed tuberosity is present at the cranioventral border of the sulcus tendineus. This tubercle is considered a synapomorphy of Procellariiformes and is otherwise present only in the tropicbird *Phaethon* (see Bourdon 2005; Smith 2010). In the fossil, the tubercle is placed close to the midline as in extant albatrosses, whereas it is more cranially positioned in most petrels. The proximal end of the ulna is relatively wide with a deep incisura radialis. The marked pit which occurs between the olecranon and processus cotylaris dorsalis in many petrels appears to have been absent, though the olecranon itself has been lost.

Although the carpometacarpus is badly damaged, the intact base of the processus pisiformis indicates it was displaced towards the cranial edge of the bone and had a cranially deflected tip. The sulcus tendineus is deeply incised as in extant albatrosses, as opposed to the shallow sulcus in petrels. Metacarpal III is thin and straight, and has an obliquely flattened cross section that becomes wider distally.

The referred tarsometatarsus is similar to that of modern albatrosses in having a strongly projected eminentia intercotylaris and deep sulcus extensorius. The hypotarsal crests are damaged, but the intact bases indicate the presence of a large crista medialis flexor digitorum longus. A short platform supports three smaller crests, which bound sulci for the tendons of m. flexor hallucis longus and m. flexor perforatus digiti II. Two vascular foramina perforate the bone, the medial exiting lateral to the crista medialis flexor digitorum longus and the lateral just distal and lateral to the platform supporting the three shorter hypotarsal crests. The crista plantaris lateralis arises well distal to the level of the hypotarsal crests as in extant albatrosses, whereas in *Plotornis graculoides* this crest arises more proximally (Figure 3O–Q).

## Phylogenetic methods

In order to resolve the phylogenetic position of *Plotornis archaeonautes*, we assembled a morphological character matrix drawing on a phylogenetic study by Mayr and Smith (2012), studies of penguins that included procellariiform species as outgroups (e.g. Bertelli and Giannini 2005; Ksepka et al. 2012) and morphological work by Forbes (1882), Kuroda (1954), Matsuoka and Hasegawa (2014) and Piro (2022). We scored one representative of each extant albatross genus and seven fossil albatrosses (*Plotornis archaeonautes*, *Plotornis delfortrii, Plotornis graculoides, Tydea septentrionalis, Diomedavus knapptonensis*, the unnamed Astoria Formation albatross, and *Aldiomedes angustirostris*) and included 22 additional procellariiform species, 3 penguins and 2 loons as outgroups.

Phylogenetic analyses were conducted using PAUP*4.0a168 (Swofford 2003) with a heuristic search strategy specifying 1000 replicates of random taxon addition, with TBR branch swapping limited to 1,000,000 rearrangements per replicate. All characters were equally weighted, multistate codings were considered to represent polymorphism, and branches with a minimum length of zero were collapsed.

## Phylogenetic relationships of living and fossil albatrosses

**Figure 4.**
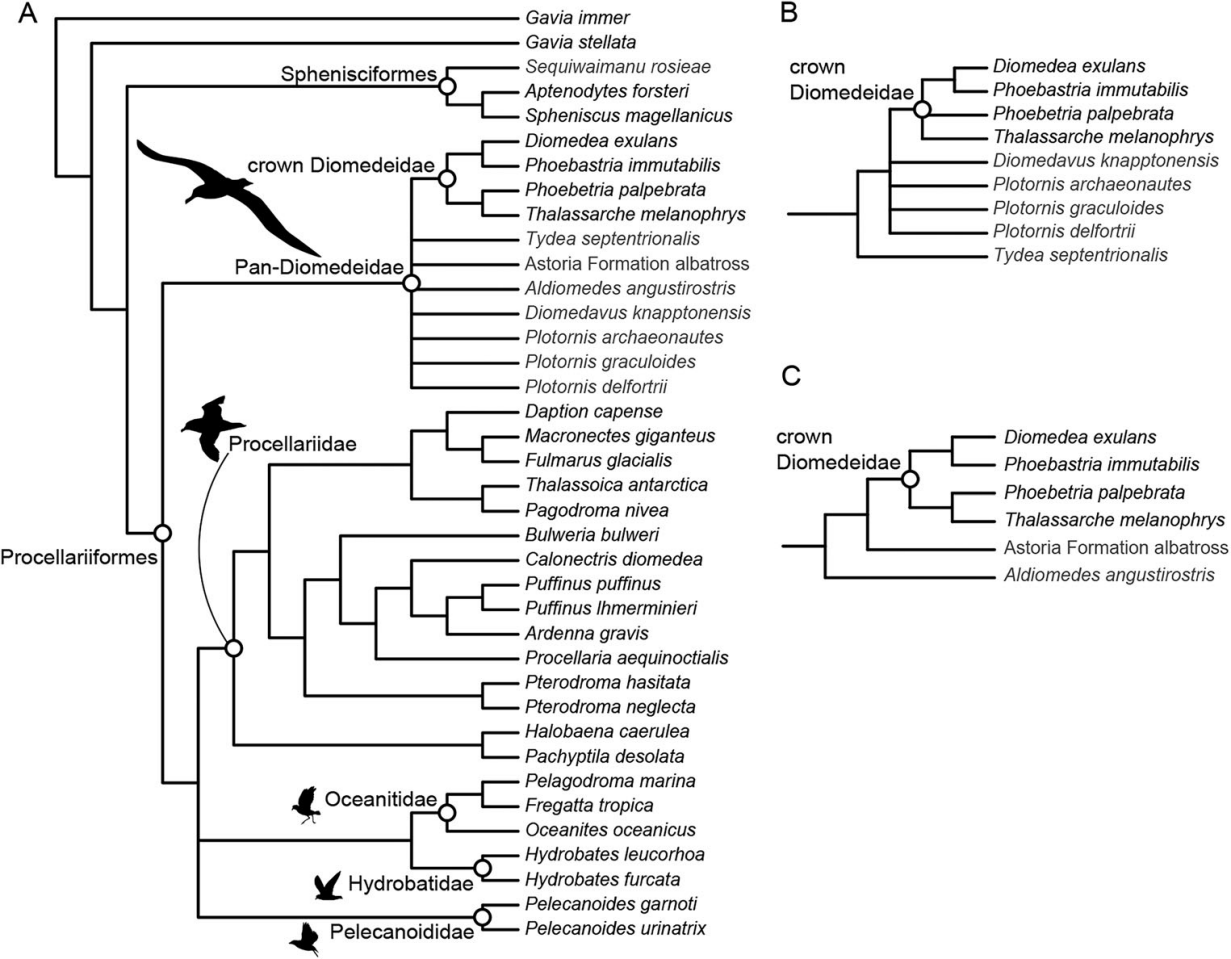
Results of phylogenetic analysis. **A**, Strict consensus of 38 MPTs of 416 steps from analysis including all taxa. **B**, Strict consensus of 4 MPTS of 416 steps from analysis excluding *Aldiomedes* and the Astoria Formation albatross. **C**, 14 MPTS of 414 steps from analysis excluding all fossil albatrosses except *Aldiomedes* and the Astoria Formation albatross. Only Pan-Diomedeidae are shown in **B** and **C**. Silhouette art from Phylopic, created by Kimberly Haddrell (Diomedeidae), Alexandre Vong (Procellariidae and Oceanitidae) and Juan Carlos Jerí (Pelecanoididae).

The initial analysis recovered 38 most parsimonious trees (MPTs) of 415 steps (Figure 4A). In the strict consensus of these trees, Diomedeidae is recovered as the most deeply branching of the extant procellariiform clades. In agreement with many previous morphological studies, the northern and southern storm petrels (Hydrobatidae and Oceanitidae) are recovered as sister taxa and the diving petrels (‘Pelecanoididae’) are recovered outside of Procellariidae (e.g. Bertelli and Giannini 2005; Livezey and Zusi 2007; Smith 2010; Ksepka et al. 2012; Mayr and Smith 2012). In contrast, most recent molecular studies nest diving petrels within Procellariidae and recover Hydrobatidae as the sister taxon to Procellariidae, rather than Oceanitidae (e.g. Ericson et al. 2006; Prum et al. 2015; Estandía et al. 2021; Kuhl et al. 2021). All fossil albatrosses were recovered within a polytomy outside of a clade uniting the four extant albatross taxa. No single character can be scored for all fossil taxa, precluding resolution of the stem albatross polytomy and unambiguous optimisation of synapomorphies.

When the Astoria Formation albatross and *Aldiomedes* (both of which lack the humerus) are excluded, *Tydea* is recovered as the earliest-branching fossil taxon, with the three species of *Plotornis* and *Diomedavus* recovered one node crownward as part of a polytomy (Figure 4B). Relationships outside Pan-Diomedeidae are identical to those recovered in the primary analysis.

Two character states optimise as unambiguous synapomorphies of Pan-Diomedeidae: (76:0) presence of pneumatic foramina within the fossa pneumotricipitalis and (114:2) a deep sulcus tendineus. Many other potential synapomorphies of Pan-Diomedeidae are ambiguous due to missing data in *Tydea* but optimise as diagnostic for the clade under an accelerated transformation model. These include (27:1) strong posterior projection of the lacrimal into the orbital region, (36:1), wide midline separation of the fossae temporales; (50:1) absence of a processus ventralis in the thoracic vertebrae, (65:1) strong ventral deflection of the omal end of the coracoid; (68:1) presence of pneumatic foramina in the impressio musculi sternocoracoidei; (75:0) sulcus transversus forming a shallow, ovoid depression; (83:0) capital shaft ridge contacting caput humeri close to the midline and (125:1) tuberculum m. gastrocnemius lateralis well-developed.

A clade uniting all other albatrosses to the exclusion of *Tydea* is supported by (79:1) presence of a marked cranial bulge on the crista bicipitalis. One character, (87:2) a moderately developed processus supracondylaris dorsalis, supports a position for *Plotornis* both crownward of *Tydea* (in which the process is weak) and outside of crown Diomedeidae (in which it is strongly projected).

The monophyly of crown Diomedeidae to the exclusion of all sampled fossils is supported unambiguously by (16:1) presence of a dorsal bulge at the base of the beak (see Mayr and Goedert 2017) and ambiguously by (91:1) a squared distal end of the processus supracondylaris dorsalis. Within crown Diomedeidae, a sister group relationship between *Diomedea* and *Phoebastria* is supported by (42:1) presence of a pneumatic foramen on the caudal face of the processus oticus of the quadrate and (48:1) presence of a pneumatic foramen within the cotyla medialis of the mandible. A sister group relationship between *Thalassarche* and *Phoebetria* is supported by (132:1): extension of the crista fibularis distal to the level of the nutrient foramen on the tibiotarsus shaft (see Matsuoka and Hasegawa 2014.)

Although the precise placement of *Aldiomedes* and the Astoria Formation albatross could not be established due to the lack of postcranial material, a supplementary analysis including only these two taxa recovered the Astoria Formation albatross as closer to the crown clade based on two features: (18:1) large bony nares and (21:0) wide pila supranasalis. Two primitive features also indicate a stem placement for *Aldiomedes*, though they cannot be scored for the Astoria Formation albatross: (37:1) moderately deep fossae temporales and (44:0) lack of a midline spur of bone underlying the mandibular symphysis.

## Wing ossifications in albatrosses

Albatrosses are arguably the most efficient soaring birds in existence. They are capable of travelling thousands of kilometres with minimal energy expenditure via dynamic soaring. In this mode of flight, birds repeatedly pass through the boundary layer above ocean waves, climbing upwind and diving downwind, and thus harvesting energy from the differential in wind speed. One of the most important anatomical innovations that aid albatrosses in soaring is a ‘shoulder-lock’ mechanism that allows them to passively hold their wings in extended position. However, this is achieved by an internal tendon of m. pectoralis whose development cannot be inferred from osteological specimens (Meyers and Stakebake 2005). A second derived feature related to soaring is the presence of two ossifications associated with the propatagial ligaments and extensor muscles of the wing. Meckel (1825) described these elements, and Reinhardt (1873) elaborated on their presence or absence in various procellariiform taxa. The smaller of these two ossifications is a flattened ovoid sesamoid which forms in the dorsal of the two tendons of origin of m. extensor carpi radialis. Reinhardt (1873) noted that this sesamoid is lacking in some albatrosses, which appears to be the case in one specimen of *Thalassarche cauta* that we examined (OM AV10444: Figure 3N). A much larger, irregular shaped ossification is anchored both to the small sesamoid and to the ventral base of the processus supracondylaris dorsalis by a tendon associated with the ventral origin of m. extensor carpi radialis, and attaches distally to the ligamentum propatagiale (Forbes 1882) (Figure 3R). This ossification does not appear to form directly within an individual ligament or tendon but rather at the nexus of several, and thus we avoid referring to it as a sesamoid. It is absent in all Hydrobatidae and Oceanitidae, but occurs in a few members of Procellariidae (e.g. *Puffinus, Calonectris, Pterodroma*) (Brooks 1937). Forbes (1882) noted that a tendonous band from m. deltoideus, pars protagialis furthermore attaches to the ligament that connects the small sesamoid and the larger ossification.

Brooks (1937, p. 82) appears to be the first to have deciphered the functional significance of the patagial element, which he referred to as the ‘spreader bone’, noting that he ‘ … drew the attention of Mr. Gregory Mathews … to this special feature of its wing structure’. Mathews (1935, 1936) coined the name moklosteon (presumably from the Greek indicating ‘lever bone’) for the element. He referred to the smaller sesamoid as the ossicle, and to the ligament connecting them as the ‘sanosteon’. These three together, along with the processus supracondylaris dorsalis, Mathews (1935) considered to form the ‘os obex’, which he and Brooks (1937) concluded serves as a strut to support the ligamentum propatagiale, essentially bracing the patagial fan like a spar. The terms ‘spreader bone’ and ‘moklosteon’ failed to gain traction in the literature, and do not to our knowledge appear in subsequent works. In the interests of standardising terminology, we suggest the name os sesamoideum m. extensoris carpi radialis for the smaller ossification. We informally refer to the larger ossification as the patagial ossicle pending further investigation of its development.

As of yet, neither element has been documented in fossil albatrosses. Milne-Edwards (1874) concluded that because the processus supracondylaris dorsalis of *Plotornis delfortrii* lacked a flattened distal edge, this species lacked the patagial ossicle. Mayr and Smith (2012) considered the enlargement of the processus supracondylaris dorsalis in modern albatrosses to correspond to the development of the patagial ossicle, and considered the weak projection of the process in *Tydea septentrionalis* may indicate less proficient dynamic soaring abilities compared to extant species. In *Plotornis archaeonautes*, a well-developed scar on the dorsal face of the processus supracondylaris dorsalis corresponds to the origin of the ligament tethering the sesamoideum m. extensoris carpi radialis to the humerus. We thus speculate that at least one of the ossifications contributing to the patagial bracing system developed in extant albatrosses was present in *Plotornis*. Extrapolating the development of the patagial ossicle is more difficult. Some taxa with a well-developed processus supracondylaris dorsalis lack both the os sesamoideum m. extensoris carpi radialis and the patagial ossicle (e.g. *Macronectes giganteus;* Figure 3H). In addition, the attachment of the patagial ossification differs in albatrosses (where it attaches via a ligament to either the sesamoideum m. extensoris carpi radialis or to the dorsal base of the processus supracondylaris dorsalis) and in petrels (in which it attaches to the tip of the processus supracondylaris dorsalis via a piece of cartilage when present). Thus, the lack of the flattened distal margin of the processus supracondylaris dorsalis does not rule out presence of the ossicle (contra Milne-Edwards 1874).

## Discussion

*Plotornis archaeonautes* provides the first insight into the cranial morphology of the small stem albatross genus *Plotornis*. Mayr and Tennyson (2020) interpreted the more slender beak of the Pliocene stem albatross *Aldiomedes angustirostris* as evidence for a more fish-heavy diet as opposed to extant albatrosses which rely heavily on squid. A similarly slender beak in the earliest Miocene *Plotornis archaeonautes* suggests such a diet may have been more widespread in stem albatrosses, though Mayr and Tennyson (2020) already cautioned that other Miocene albatrosses have widened beaks and so the narrow condition cannot be assumed to be ancestral. *Plotornis* albatrosses managed to achieve a very wide distribution, which with the addition of *Plotornis archaeonautes* and undescribed South Carolina material now encompasses all four hemispheres. Because their basal metabolic costs make soaring less efficient in terms of maximum range, many smaller procellariiformes expend large amounts of energy in active flapping flight, trading higher energetic costs for higher speed (Pennycuick 1987). In contrast, albatrosses travel primarily by soaring along waves, travelling at lower speeds than powered flight but covering much larger maximum ranges by expending proportionately less energy (Pennycuick 1987). *Plotornis archaeonautes* appears to have overlapped in size with the smallest extant albatrosses, and so assuming a similar feathered wing shape, this species was likely capable of efficient travelling over wide areas of open ocean.

## Data Availability

Character descriptions and a NEXUS file containing the matrix used in the phylogenetic analyses are openly available in figshare at 10.6084/m9.figshare.24175026.v1.
